# Bivariate Copula‐Based Regression for Joint Modeling of Healthcare Visits

**DOI:** 10.1002/hec.70059

**Published:** 2025-11-15

**Authors:** Giampiero Marra, Rosalba Radice

**Affiliations:** ^1^ Department of Statistical Science University College London London UK; ^2^ Bayes Business School, City St George’s University of London London UK

**Keywords:** additive predictor, copula regression, count data, dependence, healthcare utilization data, prediction, unobserved heterogeneity

## Abstract

Doctor and non‐doctor visit frequencies are key indicators of healthcare access, utilization and individual health‐seeking behavior. While doctor visits reflect engagement with formal medical services, non‐doctor visits, such as to nurses, physiotherapists or alternative providers, offer insights into patient preferences and system adaptability. Modeling these outcomes separately can hide relevant interdependencies and hence lead to incomplete conclusions. To address this, we employ a copula additive distributional regression framework to jointly model doctor and non‐doctor visits as flexible functions of demographic, socioeconomic and health‐related covariates. The estimation approach allows all the distributional parameters, including location, scale and the dependence structure, to vary with covariates via additive predictors. Application of the model to data from the 2012 Medical Expenditure Panel Survey reveals key determinants of physician and non‐physician visits, such as age, income and health status. Importantly, the method allows for the modeling of shared unobserved heterogeneity and effectively captures how changes in one type of utilization influence the other, thereby yielding a deeper understanding of healthcare behavior.

## 1 Introduction

Healthcare utilization patterns, particularly the frequency of doctor and non‐doctor visits, play a crucial role in shaping health outcomes, healthcare costs and policy decisions. According to the World Health Organization, ensuring timely access to essential healthcare services is crucial for improving health outcomes and preventing the progression of avoidable diseases, as part of efforts to achieve Universal Health Coverage (World Health Organization 2025). While doctor visits have traditionally been the primary metric for assessing healthcare utilization, non‐doctor visits, such as those to nurses, physiotherapists and complementary or alternative healthcare providers, are increasingly recognized as essential components of modern healthcare systems. Recent studies highlight this shift: a 2024 analysis by the National Institutes of Health reported that the use of complementary health approaches among U.S. adults rose from 19.2% in 2002 to 36.7% in 2022, particularly for pain management and preventive care (Nahin et al. 2024).

Despite the importance of understanding the dynamics of both physician and non‐physician visits, studies that explicitly model these two types of healthcare utilization jointly remain limited (Gurmu and Elder 2000; Hofer and Leitner 2012). Copula regression models are particularly useful in this context, as they can capture the dependence between outcomes, for instance induced by common latent variables, while providing flexibility in modeling each marginal distribution (Nelsen 2006; Joe 2014). In particular, copula count data models, widely applied in fields such as insurance, economics and biomedical research (Famoye and Consul 1995; Cameron et al. 2004; Famoye 2010; Gurmu and Elder 2012; Ma et al. 2020; Cho et al. 2023), offer a flexible framework for modeling associations arising from shared unobserved heterogeneity in count outcomes and were first proposed by van Ophem (1999). Importantly, this modeling strategy treats both types of visits on equal footing, thereby accounting for the fact that they represent distinct yet complementary aspects of healthcare utilization.

This article employs the copula additive distributional regression model of van der Wurp et al. (2020) to jointly analyze doctor and non‐doctor visits. This framework enables the joint modeling of overdispersed count outcomes, while allowing all the distributional parameters, including location, scale and the copula dependence coefficient, to vary with covariates through structured additive predictors. It accommodates a broad spectrum of distributions and covariate effects (including linear terms, nonlinear smooth functions, spatial components and random effects), thereby supporting the nuanced modeling of complex relationships. This flexibility is particularly valuable in healthcare utilization studies, where both marginal behaviors and their interdependence may vary across individual, contextual or system‐level factors, features often overlooked by existing bivariate count models. By capturing these dimensions simultaneously, the model can inform more targeted interventions and efficient healthcare resource allocation.

An important feature of the employed framework is that it is at once highly flexible and explicitly parametric. This duality is valuable: the parametric formulation encourages the systematic exploration of competing functional forms, facilitates the empirical evaluation of substantive hypotheses and enables the straightforward computation of interpretable, model‐based statistics. Some might rightly regard parametric assumptions as restrictive, since using an unsuitable joint distribution may lead to misleading results. However, the modular structure of the methodology affords substantial leeway in model specification (covering a wide range of distributions, covariate effects and dependence structures) and can be readily extended to alternative distributions if warranted, thereby mitigating such a concern. In this sense, the approach captures much of the spirit of distribution‐free approaches, insofar as the data are allowed to guide the selection of meaningful structures. This perspective resonates with the view articulated by Sir David R. Cox and others, who emphasized that the value of parametric models in empirical research is often overlooked (Reid 1994).

The empirical analysis examines physician and non‐physician visit data from the 2012 Medical Expenditure Panel Survey (MEPS), with the aim of elucidating detailed patterns in marginal, joint and conditional aspects of healthcare utilization. Joint modeling is particularly important due to the complex dependencies that often exist between these outcomes. Specifically, factors such as health‐seeking behavior, preferences and lifestyle may influence decisions to seek care from both provider types, generating associated patterns of use; ignoring this dependence can lead to misleading inferences and suboptimal policy recommendations. Simultaneous modeling physician and non‐physician visits accounts for shared latent heterogeneity arising from these factors, yielding a clearer and more comprehensive understanding of healthcare‐seeking behavior.

The application of copula additive distributional regression models to healthcare utilization data represents a significant step toward understanding the multifaceted dynamics of healthcare access and improving system‐level efficiency. Specifically, in addition to estimating covariate effects on the marginal and dependence parameters of the copula model, which already provides valuable insights, the methodology yields a range of joint and conditional model‐based statistics that are directly interpretable in a health policy context. These measures include joint and conditional probabilities, as well as conditional expectations, allowing for a detailed characterization of healthcare utilization. For instance, the framework allows for the evaluation of conditional probabilities, such as the likelihood of zero doctor visits given the number of non‐doctor visits, or vice versa, highlighting how engagement with one type of service affects the other across patient profiles defined by age, income and other covariates. It also provides conditional expectations, illustrating the positive association between doctor visits and non‐doctor visits. The strongest dependence occurs at low‐to‐moderate visit counts, followed by a plateau at higher levels, and the slope is steeper for doctor visits conditional on non‐doctor visits than the reverse, reflecting asymmetry in utilization patterns. These findings have clear implications for healthcare planning and policy. The positive association and asymmetry suggest that high utilization in one domain signals increased demand in the other, supporting integrated service delivery models, such as team‐based or co‐located care. The plateau at higher counts indicates that staffing and resource allocation can be focused on patients with low‐to‐moderate visit frequencies, where demand is most variable. Conditional patterns across age, income and other covariates reveal disparities in access and utilization, pointing to the need for targeted interventions, such as outreach to underserved populations, preventive care programs and culturally tailored education.

Alternatively, a quasi‐Poisson regression approach could be used, modeling the two responses separately rather than jointly. In this case, two distinct regression equations would be specified, each including the other outcome as a covariate: one for doctor visits with non‐doctor consultations as a predictor, and one for non‐doctor visits with doctor consultations as a predictor. This strategy is attractive in that it flexibly handles overdispersion and provides interpretable covariate effects on expected counts, while not requiring a full distributional assumption. However, such a method can not explicitly account for the dependence between the outcomes that may arise from shared unobserved heterogeneity, treats the responses asymmetrically instead of placing them on equal footing, and cannot yield model‐based statistics such as joint and conditional probabilities, features that also contribute to the analysis considered in this paper. In other words, a quasi‐Poisson specification would yield empirical results of narrower scope, particularly with respect to joint utilization dynamics and conditional patterns of healthcare use.

The remainder of this paper is organized as follows. Section 2 outlines the building blocks of the adopted joint modeling approach. Section 3 discusses parameter estimation and selected model‐based statistics, followed by Section 4 which addresses inferential aspects. Section 5 presents the case study, applying the model to data from the 2012 MEPS and highlighting key findings and their implications for resource allocation and decision‐making. Finally, Section 6 provides concluding remarks and outlines directions for future research. The Online Supplementary Material includes the results from a simulation study and additional case study findings.

## 2 The Model

Consider a pair of random variables for jointly modeling doctor and non‐doctor visits, $\left(Y_{1},Y_{2}\right)$, where $Y_{j}∼D_{j}(\mu_{j},\sigma_{j})$, for j=1,2, both specified using the distributions in Table 1. The parameters are defined as $\log(\mu_{j})=\eta_{\mu_{j}}(x_{\mu_{j}}\text{;}\beta_{\mu_{j}})$ and $\log\left(\sigma_{j}\right)=\eta_{\sigma_{j}}(x_{\sigma_{j}}\text{;}\beta_{\sigma_{j}})$.

**TABLE 1:** Definition and key properties of the count distributions considered in the case study. The distributional parameters μ and σ take values in (0,∞), while $y\in N_{0}$. Since the parameters must be positive, the transformation function $g_{\cdot }\left(\cdot \right)=\log\left(\cdot \right)$ is applied in all cases. Γ(⋅) is the gamma function, $\varpi =\sqrt{\frac{1}{\sigma^{2}}+\frac{2\mu }{\sigma }}$ and ${ϒ}_{\hbar }\left(\varpi \right)=\frac{1}{2}\int_{0}^{\infty }\;x^{\hbar -1}\;\exp\left\{-0.5\varpi \left(x+x^{-1}\right)\right\}dx$ is the modified Bessel function of the third kind.

Distribution	f(y;μ,σ)	E(Y)	V(Y)
Poisson (P)	$\frac{\exp\left(-\mu \right)\mu^{y}}{y!}$	μ	μ
Negative binomial type I (NBI)	$\frac{\Gamma \left(y+1/\sigma \right)}{\Gamma \left(1/\sigma \right)\Gamma \left(y+1\right)}{\left(\frac{\sigma \mu }{1+\sigma \mu }\right)}^{y}{\left(\frac{1}{1+\sigma \mu }\right)}^{1/\sigma }$	μ	$\mu +\sigma \mu^{2}$
Negative binomial type II (NBII)	$\frac{\Gamma \left(y+\mu /\sigma \right)\sigma^{y}}{\Gamma \left(\mu /\sigma \right)\Gamma \left(y+1\right){\left(1+\sigma \right)}^{y+\mu /\sigma }}$	μ	(1+σ)μ
Poisson inverse Gaussian (PIG)	${\left(\frac{2\varpi }{\pi }\right)}^{0.5}\frac{\mu^{y}\;\exp\left(1/\sigma \right){ϒ}_{y-0.5}\left(\varpi \right)}{{\left(\varpi \sigma \right)}^{y}y!}$	μ	$\mu +\sigma \mu^{2}$

The joint cumulative distribution function (CDF) of $Y_{1}$ and $Y_{2}$ is expressed as

(1) $$P\left(Y_{1}\leq y_{1},\;Y_{2}\leq y_{2}\right)=C\left(F_{1}\left(y_{1}\text{;}\mu_{1},\sigma_{1}\right),\;F_{2}\left(y_{2}\text{;}\mu_{2},\sigma_{2}\right)\text{;}\theta \right),$$

where $F_{j}(y_{j}\text{;}\mu_{j},\sigma_{j})$ is the marginal CDF of $Y_{j}$, $C:{\left(0,1\right)}^{2}\to \left(0,1\right)$ is a two‐place copula function with dependence parameter specified as $g_{\theta }\left(\theta \right)=\eta_{\theta }\left(x_{\theta }\text{;}\beta_{\theta }\right)$ and $g_{\theta }\left(\cdot \right)$ is a known monotonic one‐to‐one transformation ensuring that θ remains within its valid range. Table 2 presents the available choices for specifying the copula function. For copulae that only support positive and asymmetric dependence (e.g., Clayton and Joe), counter‐clockwise rotated versions are obtained as follows: $C_{90}\left(u_{1},u_{2}\text{;}\theta \right)=u_{2}-C\left(1-u_{1},u_{2}\text{;}\theta \right)$, $C_{180}\left(u_{1},u_{2}\text{;}\theta \right)=u_{1}+u_{2}-1+C\left(1-u_{1},1-u_{2}\text{;}\theta \right)$ and $C_{270}\left(u_{1},u_{2}\text{;}\theta \right)=u_{1}-C\left(u_{1},1-u_{2}\text{;}\theta \right)$, where the subscript of C indicates the degree of rotation, and $u_{1}$ and $u_{2}$ are the shorthand notations for the marginal CDFs used in Equation (1). The additive predictor $\eta_{\cdot }\left(x_{\cdot }\text{;}\beta_{\cdot }\right)\in R$ depends on a set of regressors $x_{\cdot }$ and parameter vector $\beta_{\cdot }$, allowing for various types of covariate effects as detailed in Section 2.1.

**TABLE 2:** Copulae considered in the case study, along with the corresponding parameter range for θ and one‐to‐one transformation function of θ. Here, $u_{1}$ and $u_{2}$ are the shorthand notations for the marginal CDFs in Equation (1), $\Phi_{2}\left(\cdot ,\cdot \text{;}\theta \right)$ denotes the CDF of the standard bivariate Gaussian distribution with correlation coefficient θ and Φ(⋅) is the CDF of the standard univariate Gaussian distribution. $t_{2,\varphi }\left(\cdot ,\cdot \text{;}\varphi ,\theta \right)$ represents the CDF of the standard bivariate Student‐t distribution with correlation θ and φ∈(2,∞) degrees of freedom, while $t_{\varphi }\left(\cdot \right)$ denotes the CDF of the standard univariate Student‐t distribution with φ degrees of freedom. Quantities $O_{1}$ and $O_{2}$ are defined as $O_{1}=1+\left(\theta -1\right)\left(u_{1}+u_{2}\right)$ and $O_{2}=O_{1}^{2}-4\theta \left(\theta -1\right)u_{1}u_{2}$, respectively.

Copula	$C\left(u_{1},u_{2}\text{;}\theta \right)$	Range of θ	$g_{\theta }\left(\theta \right)$
Ali‐mikhail‐haq (AMH)	$\frac{u_{1}u_{2}}{1-\theta \left(1-u_{1}\right)\left(1-u_{2}\right)}$	[−1,1]	$\tanh^{-1}\left(\theta \right)$
Clayton (C0)	${\left(u_{1}^{-\theta }+u_{2}^{-\theta }-1\right)}^{-1/\theta }$	(0,∞)	log(θ)
Farlie‐gumbel‐morgenstern (FGM)	$u_{1}u_{2}\left\{1+\theta \left(1-u_{1}\right)\left(1-u_{2}\right)\right\}$	[−1,1]	$\tanh^{-1}\left(\theta \right)$
Frank (F)	$-\theta^{-1}\;\log\left\{1+\left(\exp\left\{-\theta u_{1}\right\}-1\right)\right.$ $\left.\left(\exp\left\{-\theta u_{2}\right\}-1\right)/\left(\exp\left\{-\theta \right\}-1\right)\right\}$	$R\backslash \left\{0\right\}$	—
Galambos (GAL0)	$u_{1}u_{2}\;\exp\left[\left\{{\left(-\log u_{1}\right)}^{-\theta }\right.\right.$ $\left.{\left.+{\left(-\log u_{2}\right)}^{-\theta }\right\}}^{-1/\theta }\right]$	(0,∞)	log(θ)
Gaussian (*N*)	$\Phi_{2}\left(\Phi^{-1}\left(u_{1}\right),\Phi^{-1}\left(u_{2}\right)\text{;}\theta \right)$	[−1,1]	$\tanh^{-1}\left(\theta \right)$
Gumbel (G0)	$\exp\left[-\left\{{\left(-\log u_{1}\right)}^{\theta }\right.\right.$ $\left.{\left.+{\left(-\log u_{2}\right)}^{\theta }\right\}}^{1/\theta }\right]$	[1,∞)	log(θ−1)
Joe (J0)	$1-\left\{{\left(1-u_{1}\right)}^{\theta }+{\left(1-u_{2}\right)}^{\theta }\right.$ ${\left.-{\left(1-u_{1}\right)}^{\theta }{\left(1-u_{2}\right)}^{\theta }\right\}}^{1/\theta }$	(1,∞)	log(θ−1)
Plackett (PL)	$\left(O_{1}-\sqrt{O_{2}}\right)/\left\{2\left(\theta -1\right)\right\}$	(0,∞)	log(θ)
Student's *t* (T)	$t_{2,\varphi }\left(t_{\varphi }^{-1}\left(u_{1}\right),t_{\varphi }^{-1}\left(u_{2}\right)\text{;}\varphi ,\theta \right)$	[−1,1]	$\tanh^{-1}\left(\theta \right)$

The main practical advantage of copulae is that, given arbitrary marginal CDFs and a copula function linking them, it is possible to construct a multivariate distribution from an otherwise difficult‐to‐define joint CDF. Another key benefit of the copula approach is that the selection of marginal distributions and the dependence structure can be treated as separate but related aspects, which aids in model building. A potential challenge arises when one or both margins are not continuous, as this can affect the identifiability of the copula function. However, as noted by several authors (e.g., Trivedi and Zimmer 2007; Yang et al. 2020), this issue is generally not a concern in a regression context with continuous covariates: such regressors expand the ranges of $F_{1}\left(y_{1}\text{;}\mu_{1},\sigma_{1}\right)$ and $F_{2}\left(y_{2}\text{;}\mu_{2},\sigma_{2}\right)$ from discrete points to continuous intervals, ensuring the copula is uniquely determined within the region defined by their possible values.

### 2.1 Additive Predictor

For notational simplicity, let us consider an arbitrary $\eta_{i}$. The key advantages of using additive predictors are that various types of covariate effects can be dealt with, and that such effects can be flexibly determined from the data without making a priori assumptions regarding their forms (Wood 2017).

An additive predictor can generically be defined as

$$\eta_{i}=\beta_{0}+\sum_{k=1}^{K}s_{k}\left(r_{ki}\right),$$

where $\beta_{0}\in R$ is an overall intercept, $r_{ki}$ denotes the $k^{th}$ sub‐vector of the complete vector $r_{i}$, given by the union of $x_{i\mu_{1}}$, $x_{i\mu_{2}}$, $x_{i\sigma_{1}}$, $x_{i\sigma_{2}}$ and $x_{i\theta }$, and each of the K functions is represented as a linear combination of $J_{k}$ basis functions $b_{kj_{k}}\left(r_{ki}\right)$ and regression coefficients $\beta_{kj_{k}}\in R$, i.e. $\sum_{j_{k}=1}^{J_{k}}\beta_{kj_{k}}b_{kj_{k}}\left(r_{ki}\right)$. The vector of evaluations ${\left\{s_{k}\left(r_{k1}\right),\text{…},s_{k}\left(r_{kn}\right)\right\}}^{T}$ can be written as $R_{k}\beta_{k}$ with $\beta_{k}={\left(\beta_{k1},\text{…},\beta_{kJ_{k}}\right)}^{T}$ and design matrix $R_{k}\left[i,j_{k}\right]=b_{kj_{k}}\left(r_{ki}\right)$. The $s_{k}\left(\cdot \right)$ terms are subject to centering constraints which are imposed using the approach by Wood (2017). Each $\beta_{k}$ has a related quadratic penalty $\lambda_{k}\beta_{k}^{T}S_{k}\beta_{k}$ which is needed during model fitting to enforce specific properties on the $k^{th}$ function, such as smoothness. Smoothing parameter $\lambda_{k}\in \left(0,\infty \right)$ controls the trade‐off between fit and smoothness, whereas $S_{k}$ only depends on the chosen spline basis. The overall penalty can be defined as $\beta^{T}S_{\lambda }\beta$, where $\beta ={\left(\beta_{0},\beta_{1}^{T},\text{…},\beta_{K}^{T}\right)}^{T}$, $S_{\lambda }=0⊕\lambda_{1}S_{1}⊕\text{⋯}⊕\lambda_{K}S_{K}$, ⊕ denotes the direct sum operator and $\lambda ={\left(\lambda_{1},\text{…},\lambda_{K}\right)}^{T}$. The above formulation allows for many types of covariate effects (e.g., non‐linear, spatial Markov random field, smooth interactions). In fact, several definitions of basis functions and penalty terms are supported by the GJRM R package (Marra and Radice 2025) which implements the adopted model. These definitions are based on Wood (2017) to which the reader is referred for a thorough discussion. The following sections outline the types of effects used to specify the model equations in the case study.

#### 2.1.1 Effects of Binary and Factor Variables

In such cases, $s_{k}\left(r_{ki}\right)=r_{ki}^{T}\beta_{k}$, where the design matrix is constructed by stacking all covariate vectors $r_{ki}$ into $R_{k}$. Typically, such effects do not have penalties applied to them, therefore $S_{k}=0$.

#### 2.1.2 Nonlinear Effects

These involve continuous covariates, such as age, and can be flexibly determined from the data using the popular penalized regression spline approach. The main requirement is a global smoothness assumption regarding differentiability. For a continuous variable $r_{ki}$, the design matrix $R_{k}$ contains the evaluations of the $J_{k}$ known spline basis functions $b_{kj_{k}}\left(r_{ki}\right)$ for each i. To enforce smoothness, a conventional and theoretically sound choice is $S_{k}=\int m_{k}\left(r_{k}\right)m_{k}{\left(r_{k}\right)}^{T}dr_{k}$, where the $j_{k}^{th}$ element of $m_{k}\left(r_{k}\right)$ is given by $\partial^{2}b_{kj_{k}}\left(r_{k}\right)/\partial r_{k}^{2}$ and the integration is over the range of $r_{k}$. This approach can accommodate various definitions of basis functions and penalties (e.g., penalized cubic regression and B‐splines).

When setting up the basis functions, the type of spline, $J_{k}$ and, in most cases, knots need to be specified. For one‐dimensional smooth terms, the specific choice of spline basis does not usually affect the results. $J_{k}$ is typically set to 10 as this value offers sufficient flexibility in most applications. However, analyzes with larger values can be conducted to assess the sensitivity of the smooth estimates to $J_{k}$. Regarding the selection of knots, they can be placed evenly across the values of the covariate or using its percentiles. For thin‐plate regression splines, the definition adopted in the case study, only $J_{k}$ needs to be chosen (Wood 2017).

## 3 Estimation

For a random sample ${\left(y_{i1},y_{i2},r_{i}\right)}_{i=1}^{n}$, the parameter estimator $\hat{\beta }={\left({\hat{\beta }}_{\mu_{1}}^{⊤},{\hat{\beta }}_{\mu_{2}}^{⊤},{\hat{\beta }}_{\sigma_{1}}^{⊤},{\hat{\beta }}_{\sigma_{2}}^{⊤},{\hat{\beta }}_{\theta }^{⊤}\right)}^{⊤}$ is obtained using the penalized maximum likelihood estimation approach, as detailed below.

The log‐likelihood of the count outcomes copula regression model is

$$\ell \left(\beta \right)=\sum_{i=1}^{n}\log f_{12}\left(y_{i1},y_{i2}\text{;}\mu_{i1},\mu_{i2},\sigma_{i1},\sigma_{i2},\theta_{i}\right),$$

where, dropping the subscript i for simplicity,

(2) $$\begin{array}{cccc} f_{12}\left(y_{1},y_{2}\text{;}\mu_{1},\mu_{2},\sigma_{1},\sigma_{2},\theta \right)=C\left(F_{1}\left(y_{1}\text{;}\mu_{1},\sigma_{1}\right),F_{2}\left(y_{2}\text{;}\mu_{2},\sigma_{2}\right)\text{;}\theta \right) & -C\left(F_{1}\left(y_{1}-1\text{;}\mu_{1},\sigma_{1}\right),F_{2}\left(y_{2}\text{;}\mu_{2},\sigma_{2}\right)\right) & -C\left(F_{1}\left(y_{1}\text{;}\mu_{1},\sigma_{1}\right),F_{2}\left(y_{2}-1\text{;}\mu_{2},\sigma_{2}\right)\text{;}\theta \right) & +C\left(F_{1}\left(y_{1}-1\text{;}\mu_{1},\sigma_{1}\right),F_{2}\left(y_{2}-1\text{;}\mu_{2},\sigma_{2}\right)\text{;}\theta \right) \end{array}$$

When evaluating Equation (2), $F_{j}(y_{j}-1\text{;}\mu_{j},\sigma_{j})$ is replaced with $F_{j}(y_{j}\text{;}\mu_{j},\sigma_{j})-f_{j}(y_{j}\text{;}\mu_{j},\sigma_{j})$, where $f_{j}(y_{j}\text{;}\mu_{j},\sigma_{j})$ is the $j^{th}$ marginal PMF. This adjustment is particularly relevant for the case $y_{j}=0$, where $F_{j}(-1\text{;}\mu_{j},\sigma_{j})$ has to be set to 0.

Because of the flexibility in specifying the model equations that is allowed for by the proposed modeling framework, the log‐likelihood is augmented by an overall quadratic penalty. That is,

(3) $$\ell_{p}\left(\beta \right)=\ell \left(\beta \right)-\frac{1}{2}\beta^{T}S_{\lambda }\beta ,$$

where $S_{\lambda }$, defined for all the additive predictors of the model equations, is given by $S_{\lambda ,\beta_{\mu_{1}}}⊕S_{\lambda ,\beta_{\mu_{2}}}⊕S_{\lambda ,\beta_{\sigma_{1}}}⊕S_{\lambda ,\beta_{\sigma_{2}}}⊕S_{\lambda ,\beta_{\theta }}$ and λ represents all the associated smoothing parameter vectors.

Estimation of β and λ is achieved via the efficient and stable penalized likelihood approach proposed in Marra et al. (2020), which is based on a trust region algorithm with integrated multiple smoothing parameter estimation. The trust‐region method, when supplied with the analytical score and Hessian, converges super‐linearly to a point satisfying the second‐order sufficient conditions, works well also for problems which are non‐concave or exhibit close‐to‐flat regions, and is more stable and faster compared to in‐line search methods (Nocedal and Wright 2006, Chapter 4). The method employed for the efficient and stable estimation of the smoothing parameters also requires the availability of analytical first‐ and second‐order derivatives.

The effective degrees of freedom (edf) of a model whose parameters are subject to penalization is given by $edf=\text{tr}[-H\left(\hat{\beta }\right){\left\{-H_{p}\left(\hat{\beta }\right)\right\}}^{-1}]$, where tr(⋅) is the trace operator, $\hat{\beta }$ is the estimated parameter vector, $H\left(\hat{\beta }\right)$ is the Hessian of the negative log‐likelihood at $\hat{\beta }$, and $H_{p}\left(\beta \right)=H\left(\beta \right)-S_{\lambda }$ is the penalized Hessian (e.g., Marra and Radice 2020). Equivalently, $edf=\psi -\text{tr}\left[{\left\{-H_{p}\left(\hat{\beta }\right)\right\}}^{-1}S_{\lambda }\right]$, where ψ=dim(β), which clearly shows that if λ→0 then edf→ψ, and if λ→∞ then edf→ψ−ζ, where ζ is the total number of model parameters subject to penalization. When 0<λ<∞, the model edf is equal to a value in the range [ψ−ζ,ψ]. The edf of a single smooth or penalized component is given by the sum of the corresponding trace elements.

### 3.1 Model‐Based Statistics

The expectation of doctor visits conditional on the number of non‐doctor consultations, and vice versa, can provide useful insights into the relationship between the two outcomes. For a fixed covariate vector $\tilde{r}$, selected to represent the profile of a specific individual, they are defined as

(4) $$E\left(Y_{2}|Y_{1}=y_{1},\tilde{r}\text{;}\beta \right)=\frac{1}{f_{1}\left(y_{1}\text{;}\mu_{1},\sigma_{1}\right)}\sum_{y_{2}=1}^{\infty }y_{2}f_{12}\left(y_{1},y_{2}\text{;}\mu_{1},\mu_{2},\sigma_{1},\sigma_{2},\theta \right),$$

and similarly for $E\left(Y_{1}|Y_{2}=y_{2},\tilde{r}\text{;}\beta \right)$. The estimators of these quantities are obtained by replacing β with $\hat{\beta }$.

The infinite sum in the expectations is evaluated numerically by sequentially summing over increasing values of $y_{2}$ (or $y_{1}$) until convergence of the cumulative sum is achieved. Let $C_{t}=\sum_{j=1}^{t}j\cdot f_{12}\left(y_{1},j\text{;}\cdot \right)$ denote the partial sum up to $y_{2}=t$. The summation proceeds until the relative change between successive partial sums satisfies $\left(C_{t}-C_{t-1}\right)/C_{t-1}\cdot 100<10^{-5}$. Extensive testing demonstrated that this approach produces stable and reliable estimates while maintaining computational efficiency.

In addition to conditional expectations, the modeling framework also facilitates the derivation of other relevant quantities, such as conditional probabilities $P\left(Y_{1}=y_{1}|Y_{2}=y_{2},\tilde{r}\text{;}\beta \right)$, which are obtained by taking the ratio of (2) to the marginal PMF of the conditioning variable. These measures enrich the understanding of the relationship between the responses, offering valuable insights for both statistical analysis and decision‐making.

Section 1 of the Online Supplementary Material presents the findings of a simulation study evaluating the empirical performance of the employed copula approach under both correct specification and misspecification, relative to a quasi‐Poisson model. The assessment uses the conditional expectation as the model‐based statistic of interest, since it can be obtained under both the copula regression and quasi‐Poisson frameworks. The results indicate that the copula method outperforms the quasi‐Poisson under correct specification, and delivers superior or comparable results under misspecification of the dependence structure. However, quasi‐Poisson regression is preferable when the marginal distributions are misspecified, irrespective of the dependence structure. Overall, the findings support the use of the copula approach, assuming suitable marginal distributions can be specified, as in our case study, particularly when the goal is to obtain richer insights from the modeling exercise.

## 4 Inferential Aspects

The construction of intervals draws upon the results of Wood et al. (2016) for models fitted via penalized log‐likelihoods of the general form (3). Specifically, the employed distribution is $\beta \overset{\cdot }{∼}N(\hat{\beta },V_{\beta })$, where $V_{\beta }={\left\{-H_{p}\left(\hat{\beta }\right)\right\}}^{-1}$. This result is based on the notion that penalization in estimation assumes that wiggly models are less likely than smoother ones, which translates into the prior specification $f_{\beta }\propto \exp\left\{-\beta^{T}S_{\lambda }\beta /2\right\}$. From a frequentist perspective, using $V_{\beta }$ yields close‐to‐nominal coverage probabilities because it accounts for both sampling variability and smoothing bias (Marra and Wood 2012).

For nonlinear functions of the model coefficients, intervals can be conveniently obtained by posterior simulation. For instance, a (1−ϑ)100% interval for $E\left(Y_{2}|Y_{1}=y_{1},\tilde{r}\text{;}\beta \right)$, with fixed covariate vector $\tilde{r}$, can be obtained as follows: draw V random vectors $\beta_{v}$, v=1…,V, using the distribution of β; obtain V realizations of the function of interest, $E\left(Y_{2}|Y_{1}=y_{1},\tilde{r}\text{;}\beta_{v}\right)$; calculate the (ϑ/2)‐th and (1−ϑ/2)‐th quantiles of the V realizations. Parameter ϑ is typically set to 0.05, whereas a value of V equal to 100 usually produces representative results although it can be increased if more precision is required. Note that the distribution of nonlinear functions of the model parameters need not be symmetric.

Well calibrated *p*‐values for the terms in the model are obtained using the results summarized in Wood (2017), (Section 6.12), which use $V_{\beta }$ as covariance matrix.

## 5 Healthcare Utilization

The case study uses a dataset of 10,638 observations from the 2012 MEPS, collected and published by the Agency for Healthcare Research and Quality, a division of the U.S. Department of Health and Human Services. Initiated in 1996 and ongoing, the MEPS provides one of the most comprehensive individual‐level databases on health insurance, healthcare usage, health conditions and socioeconomic characteristics.

In line with the analysis of Gurmu and Elder (2000), this study focuses on jointly modeling two associated outcomes: the number of consultations with a doctor (dvisit) and the number of visits to non‐doctor health professionals (ndvisit). These variables exhibit overdispersion, with means and standard deviations of 2.12 and 3.6 for dvisit, and 0.94 and 2.9 for ndvisit. The available covariates are reported in Table 3.

**TABLE 3:** Descriptions of covariates used in the meps data.

Variable	Description
bmi	Body mass index.
income	Income in thousands of dollars.
age	Age in years.
gender	Male = 1, female = 0.
ethnicity	1 = white, 2 = black, 3 = native american, 4 = others.
education	Education in years.
region	1 = northeast, 2 = midwest, 3 = south, 4 = west.
hypertension	1 equal to 1 if hypertension present, 0 otherwise.
hyperlipidemia	Equal to 1 if hyperlipidemia present, 0 otherwise.

Simultaneous modeling of dvisit and ndvisit is essential given their interconnected nature: individuals who frequently visit doctors may also be more likely to consult non‐doctor health professionals due to shared health conditions. Furthermore, joint modeling accounts for unobserved heterogeneity, that is, differences between individuals that are not captured by the observed covariates but that nonetheless influence healthcare use. These may include health‐related attitudes (such as proactivity in seeking care), personal preferences for different types of providers, cultural norms around healthcare utilization and unmeasured aspects of health status.

### 5.1 Model Building

The modeling approach followed a systematic process typical of copula‐based studies. Covariate selection was informed by existing literature and expert knowledge, with additive predictors allowing for nonlinear effects of the continuous covariates. Based on this, various candidate distributions for the count outcomes were explored and assessed through convergence diagnostics and residual evaluation, followed by iterative refinement. Residual analysis was based on randomized normalized quantile residuals defined as $r_{ij}=\Phi^{-1}\left(u_{ij}\right)$, for i=1,…,n and outcome j, where $u_{ij}$ is a random value drawn from the uniform distribution on $\left[F_{j}\left(y_{ij}-1\text{;}{\hat{\mu }}_{ij},{\hat{\sigma }}_{ij}\right),F_{j}\left(y_{ij}\text{;}{\hat{\mu }}_{ij},{\hat{\sigma }}_{ij}\right)\right]$. Under correct model specification $r_{ij}∼N\left(0,1\right)$, assessed via normal Q‐Q plots (Dunn and Smyth 1996). Finally, the association between the outcomes was investigated through copulae, employing various families and additive predictor configurations to determine the dependence structure with the strongest empirical support. Details are given below.

#### 5.1.1 Covariate Effects

The selection of regressors was informed by prior literature and subject‐matter expertise (see, e.g., Gurmu and Elder 2000, and references therein). Variables known to be associated with healthcare utilization were included, with their effects modeled through additive predictors incorporating smooth functions for the continuous covariates, namely bmi, income, age and education, to capture potential nonlinear relationships. The effects of the categorical variables were modeled using classical dummy variable coding, assigning a separate parameter to each category level. To preserve model parsimony and interpretability, interaction terms were not included; however, the model can readily incorporate them if specific interactions are of scientific interest or warrant consideration.

#### 5.1.2 Marginal Models and Copula Selection

For the responses, the distributions reported in Table 1 were evaluated using convergence diagnostics and residual Q‐Q plots. All models, except the one based on the Poisson distribution, exhibited satisfactory convergence and residual behavior. Among them, those based on the Negative Binomial Type II and Poisson Inverse Gaussian distributions, for doctor and non‐doctor visits, respectively, displayed the most well‐behaved residuals. The marginal models were subsequently refined. In the $\mu_{1}$ equation, for instance, the smooth term for education was replaced with a linear effect, reflecting considerations of both parsimony and plausibility. For $\sigma_{1}$, several covariates were removed (based on a 5% significance threshold), and the smooth term of bmi was replaced with a linear function, as its edf value equaled 1. Similar adjustments were applied to the additive predictors corresponding to $\mu_{2}$, $\sigma_{2}$ and θ.

The dependence structure between the marginals was specified using the copulae listed in Table 2. A Gaussian copula was first employed, with the correlation parameter θ modeled as a function of an additive predictor, analogous to the marginal regressions. Following progressive simplification of this predictor, only income and region were retained. Because the estimated correlation was consistently positive across all observations, the search for alternative dependence structures was restricted to copulae allowing only positive association. Model comparison using the Akaike Information Criterion (Akaike 1998), defined as $\text{AIC}=-2\ell \left(\hat{\beta }\right)+2edf$, pointed to the Gaussian copula as the best‐fitting option.

### 5.2 The Final Model

The selected model employs a Gaussian copula with dependence parameter modeled as $\tanh^{-1}\left(\theta \right)=\beta_{0\theta }+\beta_{1\theta }income+\beta_{2\theta }I_{region2}+\beta_{3\theta }I_{region3}+\beta_{4\theta }I_{region4}$, alongside the marginals for dvisit and ndvisit specified as

$$dvisit∼NBII\left(\mu_{1},\sigma_{1}\right)\;\text{and}\;ndvisit∼PIG\left(\mu_{2},\sigma_{2}\right),$$

where $\log\left(\mu_{1}\right)=\eta_{\mu_{1}}(x_{\mu_{1}}\text{;}\beta_{\mu_{1}})$, $\log\left(\sigma_{1}\right)=\eta_{\sigma_{1}}\left(x_{\sigma_{1}}\text{;}\beta_{\sigma_{1}}\right)$, $\log\left(\mu_{2}\right)=\eta_{\mu_{2}}(x_{\mu_{2}}\text{;}\beta_{\mu_{2}})$ and $\log\left(\sigma_{2}\right)=\beta_{0\sigma_{2}}$, with additive predictors given by

$$\begin{array}{c} \eta_{\mu_{1}}(x_{\mu_{1}}\text{;}\beta_{\mu_{1}})=\beta_{0\mu_{1}}+s_{1\mu_{1}}\left(bmi\right)+s_{2\mu_{1}}\left(income\right)+s_{3\mu_{1}}\left(age\right)+\beta_{1\mu_{1}}education\\ +\beta_{2\mu_{1}}I_{ethnicity2}+\beta_{3\mu_{1}}I_{ethnicity3}+\beta_{4\mu_{1}}I_{ethnicity4}\\ +\beta_{5\mu_{1}}I_{region2}+\beta_{6\mu_{1}}I_{region3}+\beta_{7\mu_{1}}I_{region4}+\beta_{8\mu_{1}}gender\\ +\beta_{9\mu_{1}}hypertension+\beta_{10\mu_{1}}hyperlipidemia, \end{array}$$

$$\begin{array}{c} \eta_{\mu_{2}}(x_{\mu_{2}}\text{;}\beta_{\mu_{2}})=\beta_{0\mu_{2}}+s_{1\mu_{2}}\left(bmi\right)+\beta_{1\mu_{2}}income+\beta_{2\mu_{2}}age+\beta_{3\mu_{2}}education\\ +\beta_{4\mu_{2}}I_{ethnicity2}+\beta_{5\mu_{2}}I_{ethnicity3}+\beta_{6\mu_{2}}I_{ethnicity4}\\ +\beta_{7\mu_{2}}I_{region2}+\beta_{8\mu_{2}}I_{region3}+\beta_{9\mu_{2}}I_{region4}+\beta_{10\mu_{2}}gender\\ +\beta_{11\mu_{2}}hypertension+\beta_{12\mu_{2}}hyperlipidemia, \end{array}$$

$$\begin{array}{c} \eta_{\sigma_{1}}\left(x_{\sigma_{1}}\text{;}\beta_{\sigma_{1}}\right)=\beta_{0\sigma_{1}}+s_{1\sigma_{1}}\left(income\right)+\beta_{1\sigma_{1}}bmi\\ +\beta_{2\sigma_{1}}I_{ethnicity2}+\beta_{3\sigma_{1}}I_{ethnicity3}+\beta_{4\sigma_{1}}I_{ethnicity4}\\ +\beta_{5\sigma_{1}}I_{region2}+\beta_{6\sigma_{1}}I_{region3}+\beta_{7\sigma_{1}}I_{region4} \end{array}$$

and

$$\begin{array}{c} \eta_{\sigma_{2}}\left(x_{\sigma_{2}}\text{;}\beta_{\sigma_{2}}\right)=\beta_{0\sigma_{2}}+s_{1\sigma_{2}}\left(age\right)\\ +\beta_{1\sigma_{2}}I_{ethnicity2}+\beta_{2\sigma_{2}}I_{ethnicity3}+\beta_{3\sigma_{2}}I_{ethnicity4}\\ +\beta_{4\sigma_{2}}I_{region2}+\beta_{5\sigma_{2}}I_{region3}+\beta_{6\sigma_{2}}I_{region4}+\beta_{7\sigma_{2}}gender. \end{array}$$

The $I_{.}$ terms represent indicator variables for the factor variables ethnicity and region.

**FIGURE 1:**
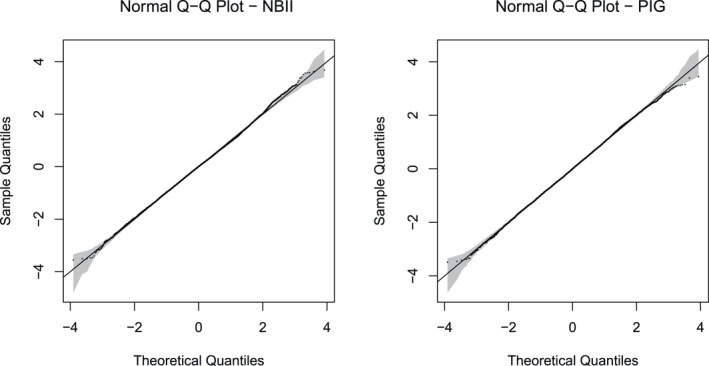
Normal Q–Q plot of randomized normalized quantile residuals, obtained after fitting a Gaussian copula additive distributional regression model with Negative Binomial II and Poisson Inverse Gaussian margins to the meps data.

At convergence, the maximum absolute gradient value was effectively zero and the observed information matrix was positive definite. The residual plots shown in Figure 1 support the chosen marginal distributions.

### 5.3 Model Fitting in R

The modeling framework is implemented in the R package GJRM (Marra and Radice 2025), which provides tools for fitting the adopted copula model and generating intuitive numerical and visual summaries. The model can be readily fitted in R as follows

library(GJRM); library(GJRM.data)

data(meps)

eq.mu1  <- dvisit  ∼ s(bmi) + s(income) + s(age) + education + ethnicity + region + gender + hypertension + hyperlipidemia

eq.mu2  <- ndvisit  ∼ s(bmi) + income + age + education + ethnicity + region + gender + hypertension + hyperlipidemia

eq.sigma1 <-  ∼ bmi + s(income) + ethnicity + region

eq.sigma2 <-  ∼ s(age) + ethnicity + region + gender

eq.theta  <-  ∼ income + region

fl <- list(eq.mu1, eq.mu2, eq.sigma1, eq.sigma2, eq.theta)

out <- gjrm(f.l, margins) = c(“NBII”, “PIG”), copula = “N”, data = meps, model = “B”, uni.fit = (TRUE)

where the various equations have the obvious interpretations, the data argument indicates the dataset used, margins defines the marginal distributions for the count responses, copula specifies the copula function employed to model the dependence between the responses and model = “B” indicates that a bivariate model is being fitted. Post‐estimation functions such as conv.check(), copula.prob(), cond.mv(), summary() and plot() are used to check for convergence and extract numerical and visual summaries, which are detailed in the next sections.

### 5.4 Marginal Results

For a typical individual (a 40‐year‐old female with a bmi of 27, an income of $47,000, 12 years of education, residing in the South, of White ethnicity and with no history of hypertension or hyperlipidemia) the estimated marginal mean for doctor visits is 1.58, with 95% interval (1.46,1.71), while for non‐doctor visits it is 0.47 (0.39,0.55). On average, this individual is expected to visit a doctor approximately 1.6 times and consult non‐doctor health professionals about 0.5 times. This disparity likely reflects a broader trend in healthcare utilization, where individuals seek care from doctors more frequently, possibly due to their perceived expertise and the central role of physicians in managing primary health concerns.

#### 5.4.1 Covariate Effects on $\mu_{1}$ and $\mu_{2}$

**FIGURE 2:**
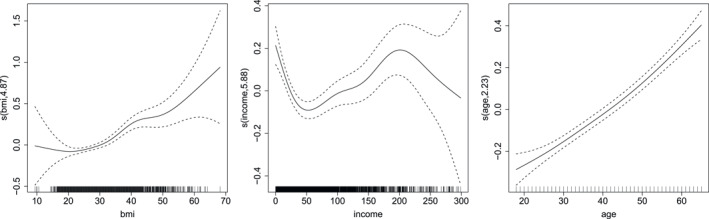
Estimated smooth effects (with associated 95% intervals) of bmi, income and age on the scale of the additive predictor of $\mu_{1}$, derived from a gaussian copula additive distributional regression model with NBI and PIG margins fitted to the meps data.

**FIGURE 3:**
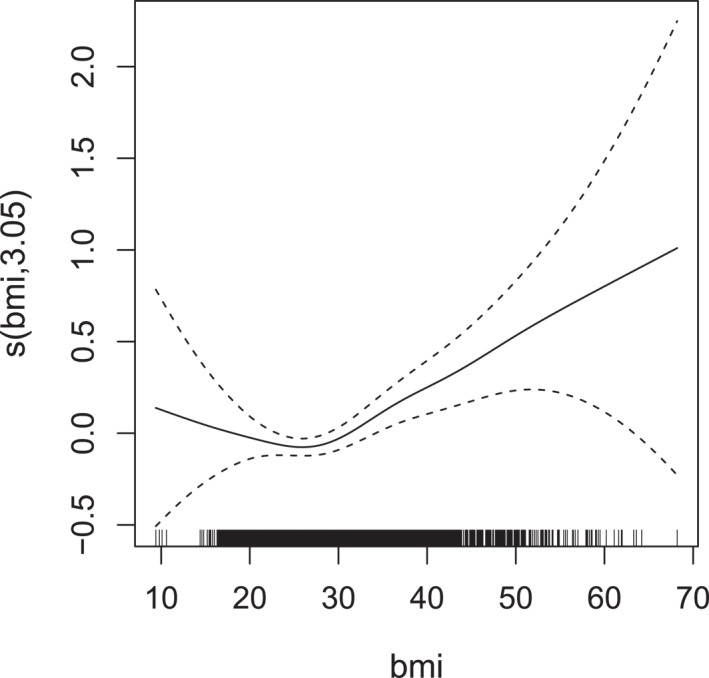
Estimated smooth effect (with associated 95% intervals) of bmi on the scale of the additive predictor of $\mu_{2}$, derived from a gaussian copula additive distributional regression model with NBI and PIG margins fitted to the meps data.

The results are summarized in Table 4 and Figures 2 and 3.

**TABLE 4:** Estimated coefficients for $\mu_{1}$ (doctor visits) and $\mu_{2}$ (non‐doctor visits), based on a Gaussian copula additive distributional regression model with NBI and PIG margins fitted to the meps data. Smooth effects for bmi, income and age are reported separately in Figures 2 and 3.

Variable	$\mu_{1}$ (doctor visits)			$\mu_{2}$ (non‐doctor visits)		
	Estimate	Std. error	*p*‐value	Estimate	Std. error	*p*‐value
(Intercept)	0.130	0.075	0.082	−3.011	0.207	<0.001
income	—	—	—	0.002	0.001	<0.001
age	—	—	—	0.013	0.003	<0.001
education	0.053	0.005	<0.001	0.178	0.011	<0.001
ethnicity2	−0.222	0.038	<0.001	−0.520	0.102	<0.001
ethnicity3	−0.106	0.144	0.459	−0.373	0.272	0.170
ethnicity4	−0.207	0.058	<0.001	−0.400	0.141	0.004
region2	0.047	0.048	0.330	0.309	0.121	0.011
region3	−0.163	0.043	<0.001	−0.406	0.118	<0.001
region4	−0.134	0.048	0.005	0.277	0.122	0.023
gender	−0.575	0.026	<0.001	−0.676	0.076	<0.001
hypertension	0.366	0.030	<0.001	0.341	0.073	<0.001
hyperlipidemia	0.444	0.030	<0.001	0.610	0.072	<0.001

The variable education exhibits a positive association with both types of visits. Specifically, each additional year of education increases expected doctor visits by about 5% (exp(0.053)≈1.054) and non‐doctor visits by nearly 20% (exp(0.178)≈1.195), suggesting that more educated individuals are more likely to seek healthcare services, particularly from non‐doctor providers. The indicator gender also plays a prominent role: males are estimated to have approximately 44% fewer doctor visits (exp(−0.575)≈0.563) and 49% fewer non‐doctor visits (exp(−0.676)≈0.509) compared to females, consistent with established patterns of higher healthcare utilization among women.

The variables age and income have small but significant linear positive effects on non‐doctor visits. Each additional year of age increases expected non‐doctor visits by about 1.3%, while each additional thousand dollars of income increases expected non‐doctor visits by approximately 0.2%. Although modest in magnitude, these effects indicate that older and higher‐income individuals are slightly more likely to consult non‐doctor healthcare professionals, which aligns with expectations that income and age influence access and utilization patterns.

Differences by ethnicity are evident as well. Compared to White individuals, Black and Other ethnicity groups exhibit lower utilization, with Black individuals having 20% fewer doctor visits and 41% fewer non‐doctor visits, and the Other group showing reductions of 19% and 33%, respectively. The effects for Native American individuals are not statistically significant, indicating similar utilization to White individuals in this sample. Variation by region is also notable: residents of the South and West regions have fewer doctor visits relative to the Northeast, whereas non‐doctor visits are higher in the Midwest and West but lower in the South. These patterns likely reflect differences in healthcare availability, local practice styles as well as cultural and behavioral factors influencing service use.

Finally, the presence of chronic conditions substantially increases healthcare utilization. Individuals with hypertension are estimated to have 44% more doctor visits and 41% more non‐doctor visits, while those with hyperlipidemia exhibit even larger increases of 56% and 84%, respectively. This is consistent with clinical expectations, as these conditions typically require regular monitoring and management by healthcare professionals.

Doctor and non‐doctor visits tend to increase with bmi, although the estimates are less precise at very low and very high values, due to the smaller number of observations in these ranges. For income, a non‐linear relationship is observed: doctor visits decline as income rises up to about $50,000, after which they gradually increase, reaching a peak around $200,000. Beyond this point, the uncertainty widens, reflecting limited data at very high levels. This non‐monotonic pattern hints at heterogeneous healthcare‐seeking behaviors across income groups and is consistent with documented non‐linearities in healthcare access and utilization. Finally, doctor visits increase with age, which is in line with expectations, as older individuals typically require more frequent medical care.

#### 5.4.2 Covariate Effects on $\sigma_{1}$ and $\sigma_{2}$

**FIGURE 4:**
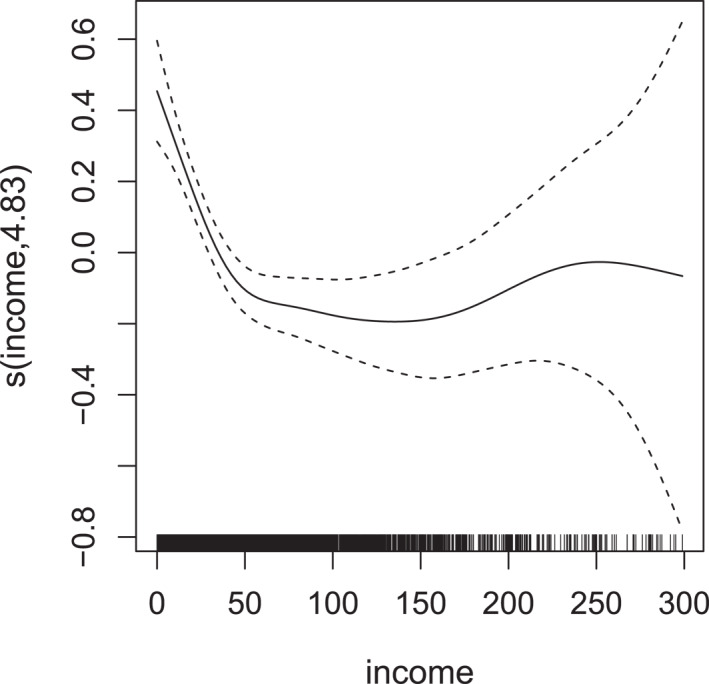
Estimated smooth effect (with associated 95% intervals) of income on the scale of the additive predictor of $\sigma_{1}$, derived from a gaussian copula additive distributional regression model with NBI and PIG margins fitted to the meps data.

**FIGURE 5:**
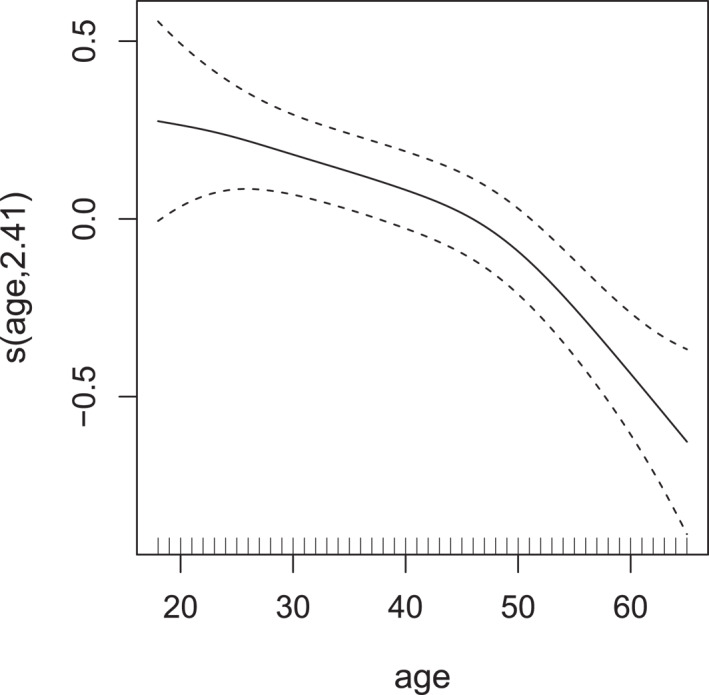
Estimated smooth effect (with associated 95% intervals) of age on the scale of the additive predictor of $\sigma_{2}$, derived from a gaussian copula additive distributional regression model with NBI and PIG margins fitted to the meps data.

A detailed overview of the results can be found in Table 5 and Figures 4 and 5.

**TABLE 5:** Estimated coefficients for $\sigma_{1}$ (doctor visits) and $\sigma_{2}$ (non‐doctor visits), based on a Gaussian copula additive distributional regression model with NBI and PIG margins fitted to the meps data. Smooth effects for income and age are reported separately in Figures 4 and 5.

Variable	$\sigma_{1}$ (doctor visits)			$\sigma_{2}$ (Non‐doctor visits)		
	Estimate	Std. error	*p*‐value	Estimate	Std. error	*p*‐value
(Intercept)	1.096	0.125	<0.001	2.483	0.149	<0.001
bmi	0.012	0.004	0.001	—	—	—
ethnicity2	−0.205	0.067	0.002	0.159	0.154	0.302
ethnicity3	−0.380	0.275	0.166	−0.922	0.530	0.082
ethnicity4	0.020	0.097	0.835	0.144	0.208	0.490
region2	−0.012	0.084	0.889	−0.375	0.177	0.034
region3	−0.189	0.076	0.013	0.024	0.175	0.892
region4	−0.034	0.083	0.680	−0.183	0.177	0.302
gender	—	—	—	0.434	0.113	<0.001

The variable bmi shows a significant positive association with the dispersion of doctor visits. This indicates that individuals with higher bmi tend to exhibit slightly greater variability in the number of doctor visits, which is consistent with the expectation that health status heterogeneity may increase as bmi deviates from typical ranges. ethnicity also plays a role in the dispersion of visits. Black individuals have slightly lower dispersion in doctor visits but slightly higher dispersion in non‐doctor visits, although the effect of the latter is not statistically significant. Other ethnic effects are generally small or non‐significant, indicating that variability across ethnic groups is less pronounced than mean differences. The effects of region on the dispersion of doctor visits are modest, with residents of the South showing slightly lower variability compared to the Northeast, while the non‐doctor visit dispersion is lower in the Midwest and largely unchanged in the South and West. The gender variable shows a notable effect for non‐doctor visits, with males exhibiting greater variability in non‐doctor consultations compared to females.

For doctor visits, the variability appears to decrease as income rises, particularly at lower levels, before reaching a plateau at higher values. This pattern is plausible, as lower‐income individuals may have more heterogeneous access to healthcare (e.g., some individuals may rarely visit a doctor due to cost or other barriers, while others may have more frequent visits due to chronic conditions) whereas higher‐income groups may exhibit more uniform utilization patterns, leading to reduced variability.

For non‐doctor visits, the variability tends to decrease with age. This observation is consistent with expectations that younger adults may show more diverse patterns of consulting non‐doctor health professionals, reflecting variation in health behaviors, preventive care use and lifestyle differences. In contrast, older individuals may have more regularized patterns of healthcare use, contributing to lower variability in non‐doctor visits.

#### 5.4.3 Covariate Effects on θ

A summary of the estimated effects is provided in Table 6.

**TABLE 6:** Estimated coefficients for the copula parameter θ, based on a Gaussian copula additive distributional regression model with NBI and PIG margins fitted to the meps data.

Variable	Estimate	Std. error	*p*‐value
(Intercept)	0.490	0.040	<0.001
Income	0.001	0.0003	0.046
region2	−0.144	0.046	0.002
region3	−0.061	0.043	0.160
region4	−0.065	0.044	0.142

The overall estimated correlation coefficient is 0.423, with 95% interval (0.372, 0.47), indicating a positive and statistically significant association between doctor and non‐doctor visits. This suggests that, after accounting for observed covariates, there remains unobserved individual‐level heterogeneity, such as health‐seeking behavior, preferences and lifestyle factors, that influences both outcomes.

Considering the covariate effects, income shows a statistically significant positive association with the copula parameter θ, indicating that the correlation between doctor and non‐doctor visits slightly increases as income rises. This pattern is plausible: higher‐income individuals may follow more consistent patterns in utilizing both types of healthcare services, resulting in stronger observed positive dependence. Part of this correlation, however, may also reflect unobserved heterogeneity. For instance, consider two individuals with similar income, age and health conditions: one may be highly proactive about preventive care, attending regular check‐ups and also consulting physiotherapists or dieticians, while the other may only seek care for acute issues. These differences in health‐seeking behavior, along with unobserved lifestyle factors such as diet, exercise and social support, can create additional association between doctor and non‐doctor visits beyond what is explained by the observed covariates. Interpreted in this context, the positive relation between income and θ suggests that such latent factors tend to affect both types of visits more consistently among wealthier individuals. In other words, high‐income individuals who are inclined to consult a doctor are also more likely to engage with non‐doctor health professionals, and this joint tendency is stronger than among lower‐income individuals. This finding reflects not only more uniform healthcare utilization in higher‐income groups, but also a tighter alignment in the ways unobserved behavioral and health‐related factors manifest across different types of healthcare services.

Regarding the region variable, compared to the Northeast, residents of the Midwest show a slightly lower correlation between doctor and non‐doctor visits, whereas the effects for the South and West are negative but not statistically significant. These patterns may partly reflect regional differences in healthcare availability, local practice norms or typical patient behavior. The modest negative effect observed for the Midwest could indicate somewhat more diverse healthcare usage patterns, while the non‐significant effects for the South and West suggest correlation levels similar to those in the Northeast.

### 5.5 Model‐Based Summaries

For the same typical individual described at the beginning of Section 5.4, joint probability estimates such as P(dvisit=0,ndvisit=0) can be computed using Equation (2). The estimated probability from the copula model is 0.446, with 95% interval (0.421, 0.470). In contrast, under the assumption of independence between the margins, the estimated probability is lower at 0.399 (0.372, 0.427).

Conditional probabilities, derived by dividing the joint probability by the marginal probability of the conditioning event, offer further insight (see Tables 7 and 8). Several patterns are immediately apparent. First, there is a consistent positive association: as $Y_{2}$ increases, the probability of observing higher counts of doctor visits also rises. For example, the probability of three doctor visits increases from 0.059 when $Y_{2}=0$ to 0.110 when $Y_{2}=3$. Similarly, the probability of two doctor visits grows from 0.099 to 0.142 across the same range of $Y_{2}$, highlighting a modest but meaningful positive dependence between the two outcomes. Second, there is a diminishing marginal effect at higher visit counts. The largest relative changes in probability occur at low‐to‐moderate levels of $Y_{2}$, while the probabilities plateau at the upper end. This is consistent with the expected distribution of healthcare utilization: extremely frequent users are rare, so incremental changes in probability are smaller at high counts. Third, the probability of zero doctor visits decreases as the number of non‐doctor visits increases (from 0.545 when $Y_{2}=0$ to 0.197 when $Y_{2}=3$), reflecting the intuitive notion that individuals engaging with non‐doctor healthcare services are less likely to have no doctor visits. Comparing these results with those obtained under the assumption of independence emphasizes the value of the copula model: under independence, the probabilities obviously remain constant across $Y_{2}$, failing to reflect the observed dependence. The copula‐adjusted probabilities thus provide a more realistic and nuanced understanding of joint healthcare utilization patterns.

**TABLE 7:** Conditional probabilities $P\left(Y_{1}=y_{1}|Y_{2}=y_{2}\right)$ with 95% intervals, where $Y_{1}$ corresponds to dvisit and $Y_{2}$ to ndvisit, derived from a Gaussian copula additive distributional regression model with NBI and PIG margins fitted to the meps data. Under the independence assumption, $P\left(Y_{1}=y_{1}|Y_{2}=y_{2}\right)=P\left(Y_{1}=y_{1}\right)$, with probabilities 0.491 (0.458, 0.522) for $Y_{1}=0$, 0.187 (0.179, 0.196) for $Y_{1}=1$, 0.107 (0.100, 0.114) for $Y_{1}=2$, and 0.067 (0.062, 0.073) for $Y_{1}=3$.

	$Y_{2}=0$	$Y_{2}=1$	$Y_{2}=2$	$Y_{2}=3$
$Y_{1}=0$	0.545 (0.519, 0.572)	0.288 (0.254, 0.320)	0.227 (0.195, 0.258)	0.197 (0.165, 0.233)
$Y_{1}=1$	0.186 (0.176, 0.195)	0.201 (0.194, 0.209)	0.188 (0.179, 0.196)	0.178 (0.168, 0.187)
$Y_{1}=2$	0.099 (0.092, 0.106)	0.140 (0.133, 0.146)	0.142 (0.135, 0.148)	0.140 (0.134, 0.146)
$Y_{1}=3$	0.059 (0.055, 0.063)	0.100 (0.094, 0.107)	0.108 (0.102, 0.114)	0.110 (0.104, 0.116)

**TABLE 8:** Conditional probabilities $P\left(Y_{2}=y_{2}|Y_{1}=y_{1}\right)$ with 95% intervals, where $Y_{1}$ corresponds to dvisit and $Y_{2}$ to ndvisit, derived from a Gaussian copula additive distributional regression model with NBI and PIG margins fitted to the meps data. Under the independence assumption, $P\left(Y_{2}=y_{2}|Y_{1}=y_{1}\right)=P\left(Y_{2}=y_{2}\right)$, with probabilities 0.814 (0.797, 0.831) for $Y_{2}=0$, 0.107 (0.098, 0.117) for $Y_{2}=1$, 0.032 (0.029, 0.035) for $Y_{2}=2$, and 0.015 (0.014, 0.017) for $Y_{2}=3$.

	$Y_{1}=0$	$Y_{1}=1$	$Y_{1}=2$	$Y_{1}=3$
$Y_{2}=0$	0.909 (0.895, 0.922)	0.816 (0.797, 0.834)	0.762 (0.740, 0.783)	0.718 (0.694, 0.742)
$Y_{2}=1$	0.061 (0.053, 0.070)	0.113 (0.102, 0.124)	0.138 (0.126, 0.150)	0.156 (0.142, 0.169)
$Y_{2}=2$	0.014 (0.012, 0.017)	0.031 (0.028, 0.035)	0.041 (0.037, 0.046)	0.049 (0.045, 0.055)
$Y_{2}=3$	0.006 (0.005, 0.007)	0.014 (0.012, 0.016)	0.019 (0.017, 0.021)	0.024 (0.021, 0.027)

As for the conditional probabilities of non‐doctor visits given the number of doctor visits, reported in Table 8, clear patterns emerge, complementing the findings in Table 7 and providing additional insight into the dependence structure between these two types of healthcare utilization. There is a strong positive association: as the number of doctor visits increases, the probability of observing higher counts of non‐doctor visits also rises. For instance, the probability of one non‐doctor visit increases from 0.061 when $Y_{1}=0$ to 0.156 when $Y_{1}=3$, while the probability of two non‐doctor visits grows from 0.014 to 0.049 across the same range. Conversely, the probability of zero non‐doctor visits decreases sharply as doctor visits increase, from 0.909 when $Y_{1}=0$ to 0.718 when $Y_{1}=3$. This pattern intuitively reflects the fact that individuals who frequently consult doctors are more likely to also engage with other health professionals. Similar to the previous table, there is a diminishing marginal effect at higher visit counts: the incremental increase in the probability of multiple non‐doctor visits becomes smaller as $Y_{1}$ rises. This plateauing is expected because extremely high counts of non‐doctor visits are rare. Finally, comparing these conditional probabilities with those obtained under the independence scenario highlights the importance of modeling dependence. Under independence, the probabilities of non‐doctor visits remain constant across levels of doctor visits, which would ignore the observed co‐movement. The copula‐based estimates, by contrast, capture this dependence effectively, providing a more realistic representation of healthcare‐seeking behavior.

Conditional expectations, as expressed in Equation (4), provide additional insight into the relationship between the two response variables. For the aforementioned individual, the conditional means are reported in Table 9.

**TABLE 9:** Conditional expectations of $Y_{1}$ given $Y_{2}$ (left) and $Y_{2}$ given $Y_{1}$ (right) with 95% intervals, derived from a Gaussian copula additive distributional regression model with NBI and PIG margins fitted to the meps data.

Y	$E\left[Y_{1}|Y_{2}\right]$	95% interval	$E\left[Y_{2}|Y_{1}\right]$	95% interval
0	1.24	(1.14, 1.35)	0.17	(0.14, 0.21)
1	2.61	(2.41, 2.84)	0.41	(0.35, 0.48)
2	3.17	(2.91, 3.52)	0.58	(0.50, 0.68)
3	3.51	(3.18, 3.85)	0.74	(0.63, 0.88)
4	3.77	(3.39, 4.18)	0.90	(0.75, 1.08)
5	3.97	(3.61, 4.43)	1.06	(0.88, 1.27)

Focusing first on doctor visits conditional on non‐doctor visits, a clear increasing trend emerges. For instance, an individual with zero non‐doctor visits is expected to have approximately 1.24 doctor visits, whereas someone with five non‐doctor visits is expected to have nearly 4 doctor visits. This pattern reflects the positive dependence captured by the copula model: individuals who frequently consult non‐doctor health professionals, such as (physiotherapists, dieticians and nurses) also tend to see doctors more often. The steepest increase occurs at low‐to‐moderate counts of non‐doctor visits, after which the conditional means continue to rise but at a slower rate, indicating a diminishing effect. This result is fully consistent with expectations, as healthcare utilization typically exhibits positive clustering at low‐to‐moderate frequencies, with saturation effects at higher counts.

Examining non‐doctor visits conditional on doctor visits, the positive association is again apparent, although the magnitude is smaller. For example, an individual with zero doctor visits is expected to have only 0.17 non‐doctor visits, while someone with five doctor visits is expected to have about 1.06 non‐doctor visits. The slope here is less steep than for doctor visits, reflecting the generally lower frequency of non‐doctor consultations. This asymmetry is intuitive: while high doctor utilization often coincides with more non‐doctor visits, the reverse effect is more pronounced because non‐doctor visits alone are less frequent, so each additional non‐doctor visit signals a stronger relative increase in doctor visits. These patterns align well with typical healthcare utilization behavior observed in population studies.

### 5.6 Broader Implications

From a policy and planning perspective, the joint modeling results provide actionable insights across multiple dimensions of healthcare utilization. The analysis reveals clear disparities in the average number of doctor and non‐doctor visits across income, gender, age and health conditions. For example, lower‐income individuals, males and certain minority groups have fewer doctor and non‐doctor visits on average, whereas visits increase with age, education and conditions such as hypertension or hyperlipidemia. These patterns suggest the need for targeted interventions to improve access for under‐served populations, such as increasing local service availability, providing culturally sensitive outreach and supporting preventive care initiatives.

The model also highlights differences in the predictability of healthcare use. The variability of doctor visits decreases with increasing income and plateaus at higher levels, while the variability of non‐doctor visits declines with age. This indicates that higher‐income individuals tend to follow more consistent doctor visit patterns, whereas older adults show more predictable non‐doctor utilization. Understanding these differences in variability can guide resource planning, for instance allocating flexible staffing to accommodate groups with high variability and streamlining services where demand is more predictable.

The positive and statistically significant copula parameter confirms that doctor and non‐doctor visits are associated. The correlation is slightly stronger among higher‐income individuals, suggesting that latent factors, such as proactive health‐seeking behavior and lifestyle habits, influence both types of visits more consistently in wealthier populations. Regional differences are modest, with Midwest residents showing a slightly lower correlation relative to the Northeast. These findings reinforce the value of coordinated, team‐based care models, where physicians and allied health professionals collaborate to address the joint patterns of utilization.

Conditional analyses show a clear link between doctor and non‐doctor visits. The probability of multiple doctor visits rises with non‐doctor visits, especially at low to moderate levels, before leveling off. On average, more non‐doctor visits are associated with more doctor visits, and vice versa, although the relationship is stronger in the direction of doctor visits given non‐doctor visits. These patterns underscore the need for integrated care planning. Doctor visits often signal downstream demand for non‐doctor services, since many care pathways begin with physician referrals. At the same time, frequent non‐doctor visits can indicate conditions that will require additional physician oversight. Recognizing this two‐way relationship allows health systems to better anticipate patient needs, align staffing across provider types and allocate resources more efficiently.

The empirical findings in this article are based on the final model fitted to the 2012 MEPS data and the characteristics of a “typical**”** individual. Similar analyze**s** using data from the 2007 and 2016 MEPS showed similar patterns (see Section 2 of the Online Supplementary Material). Section 2 also reports the conditional expectations obtained using quasi‐Poisson regression. Overall, the estimates are broadly comparable with those produced by the copula method, although some discrepancies are observed for certain values of the conditioning variables. As summarized in the final paragraph of Section 3.1, the simulation study suggests that, when appropriate marginal distributions are specified, as appears to be the case here (see Figure 1), the copula approach generally outperforms the quasi‐Poisson. Therefore, the copula‐based estimates from the case study are likely to be more reliable than those derived from the quasi‐Poisson.

## 6 Conclusions

This article employed a copula‐based additive distributional regression framework to jointly model doctor and non‐doctor healthcare visits, providing a flexible approach to address the dependence between these two forms of healthcare utilization. By allowing the parameters of the implied bivariate distribution to vary with individual‐level covariates, the approach revealed how socio‐economic characteristics and health conditions collectively influence patterns of healthcare engagement. The findings uncover meaningful behavioral trends. Importantly, the framework also yields conditional expectations and probabilities, enabling a more refined understanding of healthcare use among typical individual profiles, insights that go beyond what marginal models can offer. The empirical findings are inherently context‐specific, shaped by the structure of the data and the characteristics of the population under study. Different patterns may emerge in other settings, particularly where healthcare systems, cultural attitudes toward care and access constraints vary. Therefore, caution should be exercised when generalizing the conclusions of this paper to different populations or institutional contexts.

Despite its methodological complexity, the methodology is implemented in the freely available R package GJRM, which facilitates model estimation, visualization and interpretation. By integrating advanced statistical techniques into a user‐friendly framework, GJRM allows researchers and healthcare analysts to rigorously examine healthcare utilization patterns, such as the frequency and type of medical visits, while accounting for complex dependencies and covariate effects. The ability to generate both numerical and graphical outputs makes the tool especially useful for those seeking evidence‐based insights to inform service delivery planning, evaluate policy impacts and identify barriers to access. This can ultimately support more efficient and equitable use of healthcare resources.

Non‐physician healthcare usage is an important aspect of overall healthcare utilization. This study underscores the value of further investigating this area, both to better understand patient behavior and to inform health policy. By demonstrating a flexible and rigorous modeling approach, the work provides a foundation for future studies and encourages the field of health economics to consider the role of non‐physician care in shaping healthcare utilization patterns. In addition to this future direction, several other extensions merit exploration. Methodologically, the development of multivariate copula models that jointly analyze multiple outcomes, such as doctor visits, non‐doctor visits and emergency care, could offer a richer view of care‐seeking behavior and the related interdependencies. Substantively, the framework could also be applied beyond healthcare, to areas such as insurance claims, educational achievement and employment transitions, where related outcomes frequently arise.

## Conflicts of Interest

The authors declare no conflicts of interest.

## Supporting information


Supporting Information S1

## Data Availability

Data available through the R package GJRM.data.
