# Supplementary material for: Bivariate Copula‐Based Regression for Joint Modeling of Healthcare Visits
Source: Health Econ. 2025 Nov 15;35(2):332–45. doi: 10.1002/hec.70059 (PMC12779219; doi:10.1002/hec.70059)
Supplement: Supplementary file 1 — Supporting Information S1 [file HEC-35-332-s001.pdf]

Online Supplementary Material: Bivariate Copula-Based Regression for Joint Modeling of Healthcare Visits

Giampiero Marra, University College London, UK*

Rosalba Radice, Bayes Business School, City St George's

University of London, UK

2025-09-15

1 Simulation study

The simulation set up was based on the final model, presented in the main paper, fitted to the 2012 MEPS data. The `R` function used to simulate the responses is reported below.

```
library(copula)
library(gamlss)

data.gen <- function(eta.mu1, eta.mu2, eta.sigma1, eta.sigma2, eta.theta){
  theta.para <- tanh(eta.theta)
  Cop <- ellipCopula(family = "normal", dim = 2, param = theta.para, df = 4)
  speclist1 <- list( mu = exp(eta.mu1), sigma = exp(eta.sigma1) )
  speclist2 <- list( mu = exp(eta.mu2), sigma = exp(eta.sigma2) )
  spec <- mvdc(copula = Cop, c("NBII", "PIG"), list(speclist1, speclist2) )
  c(rMvdc(1, spec))
}
```

The `copula` `R` package contains `ellipCopula()`, `mvdc()` and `rMvdc()`, which enable simulation from the desired copula model. The `gamlss` package contains all functions required to simulate

*Corresponding author: Giampiero Marra, Department of Statistical Science, University College London, Gower Street, London WC1E 6BT, UK. E-mail: giampiero.marra@ucl.ac.uk

Negative Binomial Type II and Poisson Inverse Gaussian deviates. The various `eta` terms represent the additive predictors of the distributional parameters in the model, obtained from the final fit. These are then transformed to ensure that the restrictions on the respective parameter spaces are maintained (see Tables 1 and 2 of the main paper). Since `ellipCopula()` does not allow for the use of vectors for `param`, `data.gen()` is executed as many times as the number of observations.

The number of simulation replicates was set to 250. In each replicate, four models were fitted. Three of these were copula models: one in which both the margins and the copula were correctly specified, and two in which either the margins or the copula were misspecified. These three models were fitted using `GJRM::gjrm()`. The remaining model was a quasi-Poisson regression, fitted using `mgcv::gam()`. Each smooth function was represented using a penalized low-rank thin plate spline with a second-order penalty and 10 basis functions. In summary, the model options were:

1. **Copula** – correctly specified margins and copula.
2. **Miss.m** – misspecified marginals, using Poisson distributions.
3. **Miss.c** – misspecified dependence structure, using a Frank copula.
4. **QP** – quasi-Poisson approach, fitting a single equation with one response variable as a predictor, not requiring a full distributional assumption.

1.1 Results

Four modeling strategies for estimating the mean of y_1 conditional on y_2 were compared. The relative bias and RMSE at $y_2 = 0, \ldots, 5$ are reported in Tables 1 and 2.

Copula exhibits very small relative bias across all y_2 values ($\approx$ 1.7–2.6%), and RMSE remains low ($\approx$ 0.06–0.23), indicating high precision. Both bias and RMSE tend to increase with higher values of y_2, which is expected since larger counts are less frequent, resulting in fewer observations for accurately estimating conditional means and greater variability in the

tail. Overall, these results confirm that a correctly specified copula model provides accurate and precise estimates, even at higher y_2 values.

Miss.m shows substantial relative bias across all y_2 values, reflecting overestimation at $y_2 = 0$ and underestimation for $y_2 > 0$ of the conditional means. RMSE is also very large ($\approx$ 1.3–1.5). This demonstrates that misspecifying the margins severely compromises model performance and precision. Misspecifying the dependence structure (**Miss.c**) has a milder effect in that relative bias remains small to moderate ($\approx -15\%$ to $+8\%$) and RMSE is moderate ($\approx$ 0.09–0.63). Compared to **QP**, **Miss.c** generally achieves superior performance, with lower bias and RMSE values. The only exception is at $y_2 = 5$, where **Miss.c** and **QP** yield comparable results. In summary, **QP** performs worse than both **Copula** and **Miss.c** in almost all cases, and outperforms **Miss.m**.

	**Copula**	**Miss.m**	**Miss.c**	**QP**
$y_2 = 0$	0.017	0.349	0.055	0.167
$y_2 = 1$	0.019	-0.240	0.082	-0.332
$y_2 = 2$	0.022	-0.274	-0.028	-0.338
$y_2 = 3$	0.023	-0.258	-0.083	-0.284
$y_2 = 4$	0.025	-0.234	-0.121	-0.211
$y_2 = 5$	0.026	-0.212	-0.152	-0.132

Table 1: Relative bias of the mean estimates of Y_1 conditional of several values for Y_2 from each modeling approach.

	**Copula**	**Miss.m**	**Miss.c**	**QP**
$y_2 = 0$	0.059	1.568	0.088	0.217
$y_2 = 1$	0.118	1.328	0.247	0.872
$y_2 = 2$	0.159	1.391	0.167	1.079
$y_2 = 3$	0.188	1.498	0.327	1.014
$y_2 = 4$	0.211	1.503	0.482	0.834
$y_2 = 5$	0.230	1.514	0.625	0.613

Table 2: RMSE of the mean estimates of Y_1 conditional of several values for Y_2 from each modeling approach.

From a practical perspective, since in this work marginal distributions can be validated empirically, the most realistic scenario is one in which the copula dependence may be misspecified while the margins are correctly specified. In this case, copula-based estimation consistently

outperforms quasi-Poisson regression, demonstrating that, even with imperfect specification of the dependence structure, copula models may provide more reliable conditional mean estimates.

2 Additional case study findings

2.1 Comparison of modeling approaches in the case study

To assess the performance and differences between modeling strategies, we compare conditional mean estimates obtained from the copula model and quasi-Poisson regression applied to our case study (see Table 3 and Figure 1). This comparison highlights how each method captures the trends in the outcomes as well as the associated uncertainty in the estimates.

For the conditional means of y_1, the largest differences arise at mid-range values of y_2 ($y_2 = 1$ and $y_2 = 2$), where the quasi-Poisson estimates are noticeably lower than those from the copula model. At higher values ($y_2 = 4, 5$), the estimates from the two approaches are very similar. This pattern is consistent with the simulation results (see Table 1), where the relative bias for **Miss.c** and **QP** differs most at mid-range values of y_2, with **Miss.c** performing substantially better and **QP** systematically underestimating the means, while the biases become more similar as y_2 increases. Note that this comparison is made with respect to **Miss.c**, since the marginal distributions were assessed to be correctly specified.

		copula	quasi-Poisson
y_2	0	1.24 (1.15, 1.34)	1.34 (1.23, 1.46)
	1	2.61 (2.37, 2.84)	1.93 (1.76, 2.11)
	2	3.17 (2.89, 3.50)	2.64 (2.38, 2.93)
	3	3.51 (3.11, 3.90)	3.29 (2.95, 3.67)
	4	3.77 (3.38, 4.05)	3.72 (3.32, 4.17)
	5	3.97 (3.62, 4.30)	3.93 (3.48, 4.45)
y_1	0	0.17 (0.14, 0.21)	0.22 (0.17, 0.28)
	1	0.41 (0.35, 0.48)	0.39 (0.31, 0.48)
	2	0.58 (0.49, 0.68)	0.62 (0.49, 0.78)
	3	0.74 (0.63, 0.87)	0.84 (0.67, 1.07)
	4	0.90 (0.77, 1.08)	0.99 (0.78, 1.25)
	5	1.06 (0.87, 1.26)	1.06 (0.83, 1.35)

Table 3: Comparison of conditional mean estimates derived from the copula model and quasi-Poisson regression, together with their 95% intervals.

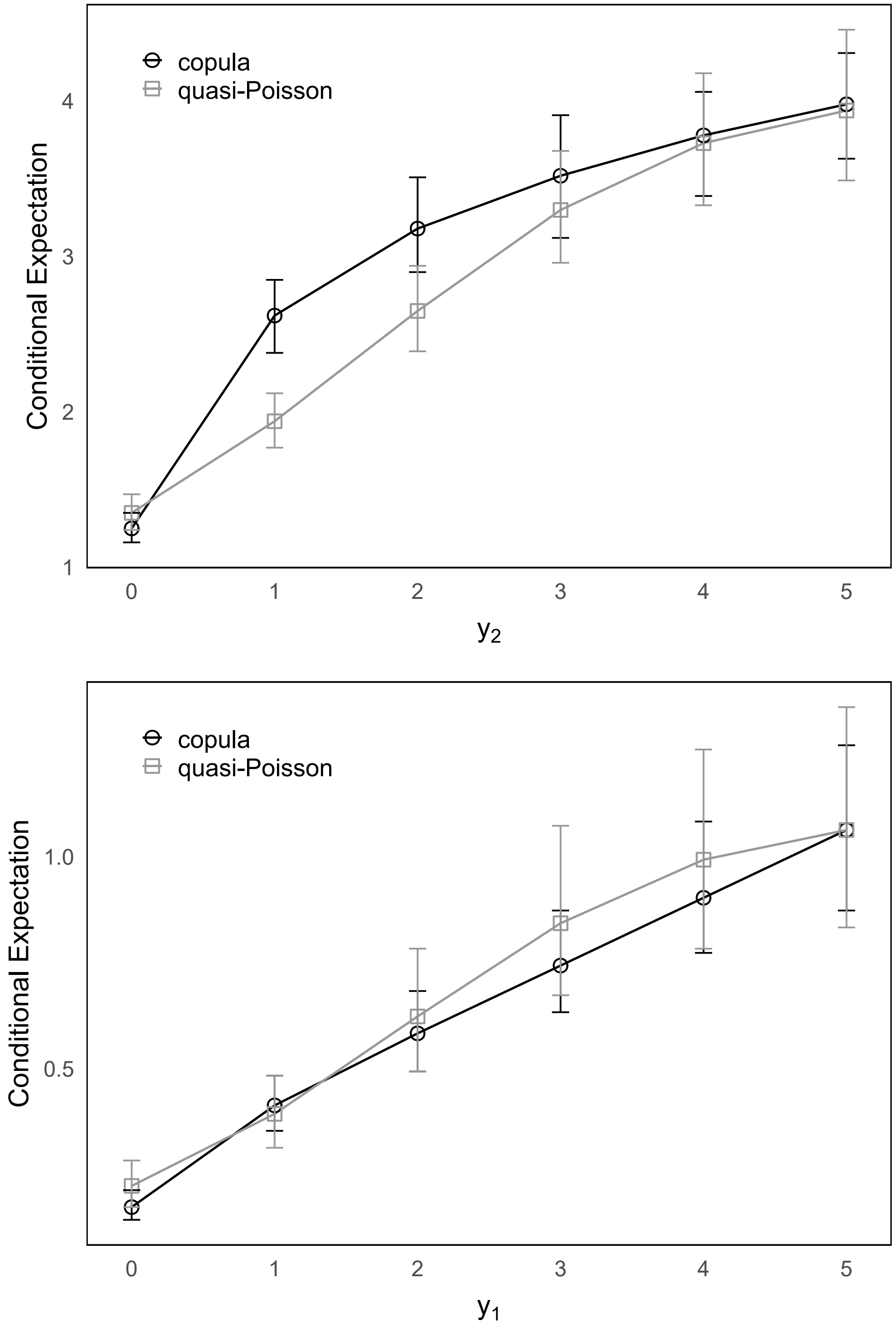

Figure 1: Estimated conditional means with 95% intervals. The top panel shows estimates of y_1 conditional on y_2, while the bottom panel shows estimates of y_2 conditional on y_1, obtained using the copula and univariate models.

2.2 Findings from MEPS 2007 and 2016

The results in this section complement the main analysis and are based on MEPS data from 2007 and 2016.

2.2.1 Results for MEPS 2007

The marginal means for `dvisit` and `ndvisit` are computed for a typical individual with the following attributes: a 40-year-old female with a `bmi` of 27, an income of $47,000, 12 years of education, residing in the South, of White ethnicity and with no history of hypertension or hyperlipidemia. The estimated marginal mean for doctor visits is 2.16, with 95% interval $(1.99, 2.35)$, while for non-doctor visits it is 0.95 $(0.84, 1.07)$. On average, this individual is expected to visit a doctor approximately 2.16 times and consult non-doctor health professionals about 0.95 times.

For the same typical individual, the estimated probability for $\mathbb{P}(\texttt{dvisit} = 0, \texttt{ndvisit} = 0)$ from the copula model is 0.339 $(0.321, 0.357)$. In contrast, under the assumption of independence between the margins, the estimated probability is lower at 0.293 $(0.276, 0.310)$. These differences underscore the importance of accounting for unobserved heterogeneity between the two outcomes.

Conditional probabilities, derived by dividing the joint probability by the marginal probability of the conditioning event, offer further insight (see Tables 4 and 5)

	$Y_2 = 0$	$Y_2 = 1$	$Y_2 = 2$	$Y_2 = 3$
$Y_1 = 0$	0.460 (0.438, 0.481)	0.251 (0.232, 0.272)	0.201 (0.183, 0.220)	0.177 (0.159, 0.195)
$Y_1 = 1$	0.239 (0.235, 0.243)	0.240 (0.234, 0.246)	0.224 (0.216, 0.233)	0.214 (0.205, 0.222)
$Y_1 = 2$	0.115 (0.110, 0.120)	0.150 (0.146, 0.154)	0.153 (0.149, 0.156)	0.152 (0.149, 0.156)
$Y_1 = 3$	0.061 (0.057, 0.065)	0.094 (0.090, 0.098)	0.101 (0.097, 0.105)	0.104 (0.100, 0.107)

Table 4: Conditional probabilities $\mathbb{P}(Y_1 = y_1 \mid Y_2 = y_2)$ with 95% intervals, where Y_1 corresponds to `dvisit` and Y_2 to `ndvisit`, derived from a copula additive distributional regression model fitted to the 2007 MEPS data. Under the independence assumption, $\mathbb{P}(Y_1 = y_1 \mid Y_2 = y_2) = \mathbb{P}(Y_1 = y_1)$, with probabilities 0.398 (0.376, 0.420) for $Y_1 = 0$, 0.234 (0.231, 0.237) for $Y_1 = 1$, 0.123 (0.118, 0.127) for $Y_1 = 2$, and 0.070 (0.067, 0.074) for $Y_1 = 3$.

	$Y_1 = 0$	$Y_1 = 1$	$Y_1 = 2$	$Y_1 = 3$
$Y_2 = 0$	0.862 (0.850, 0.874)	0.752 (0.736, 0.768)	0.684 (0.666, 0.701)	0.636 (0.616, 0.655)
$Y_2 = 1$	0.085 (0.079, 0.092)	0.137 (0.129, 0.144)	0.162 (0.154, 0.170)	0.177 (0.169, 0.185)
$Y_2 = 2$	0.023 (0.020, 0.025)	0.042 (0.039, 0.045)	0.055 (0.051, 0.058)	0.063 (0.059, 0.067)
$Y_2 = 3$	0.010 (0.009, 0.011)	0.020 (0.018, 0.022)	0.027 (0.025, 0.029)	0.032 (0.030, 0.034)

Table 5: Conditional probabilities $\mathbb{P}(Y_2 = y_2 \mid Y_1 = y_1)$ with 95% intervals, where Y_1 corresponds to `dvisit` and Y_2 to `ndvisit`, derived from a copula additive distributional regression model fitted to the 2007 MEPS data. Under the independence assumption, $\mathbb{P}(Y_2 = y_2 \mid Y_1 = y_1) = \mathbb{P}(Y_2 = y_2)$, with probabilities 0.737 (0.722, 0.752) for $Y_2 = 0$, 0.133 (0.127, 0.140) for $Y_2 = 1$, 0.044 (0.041, 0.047) for $Y_2 = 2$, and 0.022 (0.021, 0.024) for $Y_2 = 3$.

Y	$\mathbb{E}[Y_1 \mid Y_2]$	95% CI	$\mathbb{E}[Y_2 \mid Y_1]$	95% CI
0	1.58	(1.45, 1.73)	0.33	(0.29, 0.38)
1	3.04	(2.79, 3.30)	0.73	(0.65, 0.83)
2	3.69	(3.38, 4.03)	1.07	(0.95, 1.21)
3	4.08	(3.73, 4.47)	1.36	(1.21, 1.54)
4	4.37	(3.98, 4.78)	1.61	(1.41, 1.83)
5	4.60	(4.20, 5.07)	1.83	(1.61, 2.07)

Table 6: Conditional expectations of Y_1 given Y_2 (left) and Y_2 given Y_1 (right) with 95% intervals, derived from a copula additive distributional regression model fitted to the 2007 MEPS data.

2.2.2 Results for MEPS 2016

The marginal means for `dvisit` and `ndvisit` are computed for a typical individual with the following attributes: a 40-year-old female with a `bmi` of 27, an income of $47,000, 12 years of education, residing in the South, of White ethnicity and with no history of hypertension or hyperlipidemia. The estimated marginal mean for doctor visits is 1.70, with 95% interval $(1.55, 1.85)$, while for non-doctor visits it is 1.13 $(1.01, 1.27)$. On average, this individual is expected to visit a doctor approximately 1.70 times and consult non-doctor health professionals about 1.13 times.

For the same typical individual, the estimated probability for $\mathbb{P}(\texttt{dvisit} = 0, \texttt{ndvisit} = 0)$ from the copula model is 0.394 $(0.376, 0.441)$. In contrast, under the assumption of independence between the margins, the estimated probability is lower at 0.336 $(0.320, 0.353)$.

Conditional probabilities, derived by dividing the joint probability by the marginal probability of the conditioning event, offer further insight (see Tables 7 and 8)

	$Y_2 = 0$	$Y_2 = 1$	$Y_2 = 2$	$Y_2 = 3$
$Y_1 = 0$	0.561 (0.540, 0.581)	0.317 (0.294, 0.338)	0.250 (0.228, 0.272)	0.217 (0.197, 0.239)
$Y_1 = 1$	0.223 (0.217, 0.228)	0.258 (0.253, 0.263)	0.247 (0.241, 0.253)	0.237 (0.230, 0.244)
$Y_1 = 2$	0.092 (0.086, 0.097)	0.142 (0.137, 0.146)	0.150 (0.146, 0.155)	0.152 (0.148, 0.156)
$Y_1 = 3$	0.045 (0.042, 0.048)	0.082 (0.078, 0.086)	0.093 (0.089, 0.097)	0.098 (0.094, 0.101)

Table 7: Conditional probabilities $\mathbb{P}(Y_1 = y_1 \mid Y_2 = y_2)$ with 95% intervals, where Y_1 corresponds to `dvisit` and Y_2 to `ndvisit`, derived from a copula additive distributional regression model fitted to the 2016 MEPS data. Under the independence assumption, $\mathbb{P}(Y_1 = y_1 \mid Y_2 = y_2) = \mathbb{P}(Y_1 = y_1)$, with probabilities 0.480 (0.459, 0.500) for $Y_1 = 0$, 0.226 (0.223, 0.230) for $Y_1 = 1$, 0.105 (0.101, 0.110) for $Y_1 = 2$, and 0.057 (0.053, 0.060) for $Y_1 = 3$.

	$Y_1 = 0$	$Y_1 = 1$	$Y_1 = 2$	$Y_1 = 3$
$Y_2 = 0$	0.839 (0.826, 0.852)	0.688 (0.672, 0.704)	0.599 (0.578, 0.617)	0.541 (0.518, 0.561)
$Y_2 = 1$	0.099 (0.091, 0.106)	0.166 (0.158, 0.174)	0.193 (0.185, 0.201)	0.206 (0.199, 0.214)
$Y_2 = 2$	0.027 (0.024, 0.029)	0.055 (0.051, 0.058)	0.071 (0.066, 0.075)	0.080 (0.076, 0.085)
$Y_2 = 3$	0.012 (0.011, 0.013)	0.026 (0.025, 0.028)	0.036 (0.034, 0.039)	0.043 (0.040, 0.045)

Table 8: Conditional probabilities $\mathbb{P}(Y_2 = y_2 \mid Y_1 = y_1)$ with 95% intervals, where Y_1 corresponds to `dvisit` and Y_2 to `ndvisit`, derived from a copula additive distributional regression model fitted to the 2016 MEPS data. Under the independence assumption, $\mathbb{P}(Y_2 = y_2 \mid Y_1 = y_1) = \mathbb{P}(Y_2 = y_2)$, with probabilities 0.701 (0.686, 0.714) for $Y_2 = 0$, 0.147 (0.141, 0.155) for $Y_2 = 1$, 0.051 (0.048, 0.054) for $Y_2 = 2$, and 0.026 (0.024, 0.027) for $Y_2 = 3$.

Y	$\mathbb{E}[Y_1 \mid Y_2]$	95% CI	$\mathbb{E}[Y_2 \mid Y_1]$	95% CI
0	1.10	(1.00, 1.20)	0.39	(0.34, 0.43)
1	2.36	(2.17, 2.57)	0.97	(0.88, 1.08)
2	2.99	(2.73, 3.28)	1.47	(1.32, 1.64)
3	3.39	(3.07, 3.68)	1.90	(1.69, 2.13)
4	3.68	(3.36, 4.08)	2.26	(2.01, 2.57)
5	3.93	(3.56, 4.32)	2.59	(2.29, 2.93)

Table 9: Conditional expectations of Y_1 given Y_2 (left) and Y_2 given Y_1 (right) with 95% intervals, derived from a copula additive distributional regression model fitted to the 2016 MEPS data.
